# Integrative Analysis of Plasma Proteomics and Transcriptomics Reveals Potential Therapeutic Targets for Psoriasis

**DOI:** 10.3390/biomedicines13061380

**Published:** 2025-06-04

**Authors:** Hesong Wang, Chenguang Wang, Ruihao Qin, Jia He, Xuan Zhang, Chenjing Ma, Shi Li, Lijun Fan, Liuying Wang, Lei Cao

**Affiliations:** 1 Department of Biostatistics, School of Public Health, Harbin Medical University, Harbin 150086, China; 2021020186@hrbmu.edu.cn (H.W.); 2022020127@hrbmu.edu.cn (R.Q.); 15734710545@163.com (J.H.); 2021020184@hrbmu.edu.cn (X.Z.); 2022020124@hrbmu.edu.cn (C.M.); 2 Center for Endemic Disease Control, Chinese Center for Disease Control and Prevention, Harbin Medical University, Harbin 150086, China; gordon_wong0824@163.com (C.W.); fanlijun@hrbmu.edu.cn (L.F.); 3 Laboratory for the Study of Metastatic Microenvironments, Fred Hutchinson Cancer Research Center, Seattle, WA 98109, USA; sli7@fredhutch.org; 4 School of Health Management, Harbin Medical University, Harbin 150086, China; wangliuying@hrbmu.edu.cn

**Keywords:** psoriasis, plasma proteome, mendelian randomization, drug target, single-cell

## Abstract

**Background Psoriasis** (PsO)**:** is an immune-mediated inflammatory disease that imposes a significant burden on patients. Many patients experience relapse or inadequate responses, and PsO subtypes also lack effective therapies, highlighting the need for new therapeutic targets. **Methods:** We performed a proteome-wide Mendelian randomization (MR) to explore potential therapeutic targets for PsO. Protein quantitative trait loci (pQTLs) data were obtained from the Pharma Proteomics Project (54,219 UK Biobank participants, 2923 proteins), and PsO phenotype and subtype data were sourced from FinnGen (10,312 cases; 397,564 controls) for discovery. Replication MR utilized integrated protein data (Iceland and Norfolk) and phenotype data from multiple databases (UK Biobank and GWAS Catalog). Reverse MR and colocalization were used to support causal relationships. Single-cell RNA-seq analysis revealed distinct expression patterns of protein-coding genes across different cell types in PsO biopsy samples and normal skin tissues. Protein-protein interactions (PPI) and molecular docking were used to evaluate druggability. **Results:** MR analysis identified 13 proteins significantly associated with PsO risk (*p* < $2.56\times 10^{-5}$), including 10 proteins associated with PsO subtypes. Decreased levels of eight proteins (IFNLR1, APOF, TDRKH, DDR1, HLA-E, LTA, MOG, and ICAM3) and increased levels of five proteins (IFNGR2, HCG22, IL12B, BTN3A2, and TRIM40) showed protective effects against PsO progression. Robust colocalization ($PPH_{4}$ > 0.9) identified IFNLR1, IFNGR2, APOF, and TDRKH as top candidates. Single-cell RNA sequencing analysis revealed that IFNLR1, IFNGR2, LTA, TDRKH, and DDR1 were specifically expressed in T cells of psoriatic biopsy specimens compared to healthy controls. Molecular docking indicated the druggability of IFNLR1 and IFNGR2. **Conclusions:** We identified several potential therapeutic targets for PsO, with IFNLR1, IFNGR2, APOF, and TDRKH emerging as promising candidates, particularly IFNLR1 and IFNGR2, which are associated with the IFN family. These findings may provide new perspectives on PsO therapy and pathogenesis.

## 1. Introduction

PsO is a chronic immune-mediated inflammatory condition that predominantly affects the skin of the elbows, knees, scalp, and back [1]. This disease imposes significant physical and psychological burdens on patients and incurs substantial treatment costs. With a prevalence of around 2% in Europe and North America [2] and a rising incidence globally [3], PsO poses a significant health challenge. This condition is associated with an increased risk of several cancers [4,5,6], with a cancer prevalence of 4.78% among patients with PsO [7]. Additionally, individuals with PsO face a higher risk of developing various chronic conditions and health-related diseases [1]. The World Health Organization (WHO) has classified PsO as a major global health problem [8].

Current PsO treatments focus on relieving symptoms such as itching, pain, and skin lesions [9]. Despite advancements in targeted biologics, treatment outcomes remain unsatisfactory for many patients due to limited efficacy, adverse reactions, and high discontinuation rates [10,11,12]. Current treatment outcomes for PsO subtypes, such as palmoplantar PsO and pustular PsO, are unsatisfactory. Therefore, it is essential to identify more promising drug targets to address these shortcomings.

Plasma proteins are vital to human biology and disease progression and are a primary source of drug targets [13]. Protein quantitative trait loci (pQTLs) reveal associations between genetic variations and plasma protein levels, while genome-wide association studies (GWAS) establish links between genetic variations and traits. Mendelian randomization (MR) leverages these insights by using single nucleotide polymorphisms (SNPs) from protein-level GWAS as genetic instruments to estimate the causal effects of proteins on diseases. By satisfying instrumental variable assumptions [14], MR can mitigate confounding effects, providing a robust framework for exploring potential drug targets [15]. Notably, drug targets supported by replicable genetic evidence are more likely to receive regulatory approval [16]. Thus, the integration of pQTL data with the genetic associations of diseases provides significant value for drug discovery and the study of pathological mechanisms.

## 2. Materials and Methods

### 2.1. Study Design

**Figure 1:**
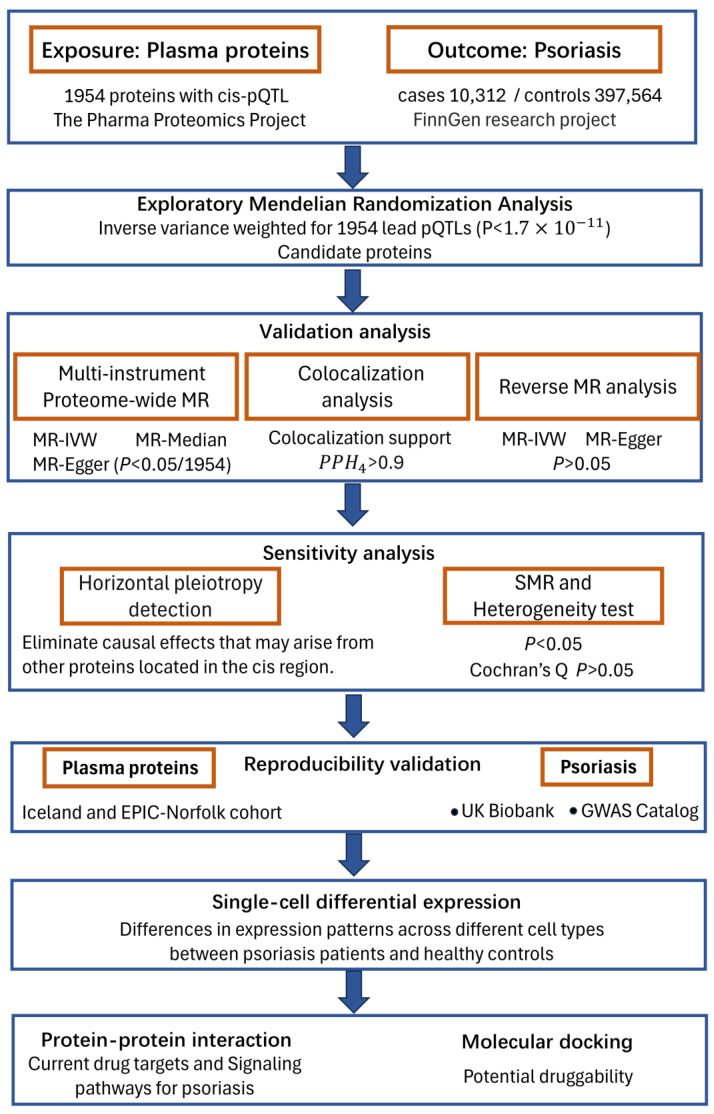
Study design for identifying causal associations between plasma proteins and PsO.

The overall study design is presented in Figure 1. This study followed the Strengthening the Reporting of Observational Studies in Epidemiology-Mendelian Randomization (STROBE-MR) Reporting Guidelines [17]. Based on the fulfillment of the three core assumptions of Mendelian randomization (relevance, independence, and exclusion restriction), causal pathways from proteins to PsO were established using stepwise analyses. While Mendelian randomization (MR) overcomes confounding and facilitates causal inference, it also has inherent limitations, such as pleiotropy and reverse causality. These issues must be addressed through appropriate strategies for practical applications. In this study, we employed rigorous methodologies to mitigate these limitations, including the use of strong instrumental variables to ensure robust associations with the disease, linkage disequilibrium filtering to reduce pleiotropy and reverse MR analysis to rule out reverse causation. First, Mendelian randomization (MR) analysis was conducted using the lead cis-pQTL (most significant *p*-value) of each protein to explore candidate proteins. To enhance the robustness of our findings, we then performed multi-instrument MR analysis, including both discovery and replication phases, on the identified candidate proteins. Further validation of the causal relationship and exclusion of potential confounding factors were achieved through reverse MR, Bayesian colocalization, heterogeneity tests, and SMR analysis. Finally, we constructed protein-protein interaction networks to map the relationships among the identified causal proteins, current drug targets, and known PsO signaling pathways to evaluate the potential of candidate proteins as viable drug targets.

### 2.2. Plasma Protein Data

The plasma pQTL data for the discovery set were obtained from the Pharma Proteomics Project in the UK Biobank [18] (2923 plasma proteins measured in 54,219 participants). For external validation, plasma pQTL data were retrieved from genome-wide association studies of plasma levels [19] (4907 plasma proteins measured in 35,559 participants) in Iceland, and the proteogenomic links study [20] from the EPIC-Norfolk cohort (2923 plasma proteins measured in 1180 participants). Proteins from the UK Biobank and EPIC-Norfolk cohort were measured using the Olink platform [18,20], while proteins from the Iceland study were measured using the SOMAscan platform [19]. The details of the plasma proteins are provided in Supplementary Table S1.

### 2.3. GWAS Summary Association Statistics of PsO

For the primary analysis, summary statistics of PsO (ICD10-L40) were derived from the FinnGen research project [21], including 407,876 participants ($n_{case}$ = 10,312, $n_{control}$ = 397,564, prevalence = 2.51%). For reproducibility validation, summary statistics of PsO were obtained from the UK Biobank ($n_{case}$ = 4815, $n_{control}$ = 415,646) [22] and GWAS Catalog ($n_{case1}$ = 5459, $n_{control1}$ = 324,074, $n_{case2}$ = 15,967, $n_{control2}$ = 28,194) [23,24,25]. Data for PsO subtypes, including guttate PsO ($n_{case}$ = 389, $n_{control}$ = 397,564), generalized pustular PsO ($n_{case}$ = 101, $n_{control}$ = 397,564), and pustulosis palmaris et plantaris ($n_{case}$ = 1249 $n_{control}$ = 397,564), were also obtained from the FinnGen project. Basic information on PsO is shown in Supplementary Table S2.

### 2.4. Mendelian Randomization (MR) Analysis

In our study, we used the R package “MendelianRandomization” (V0.10.0) to perform Mendelian randomization as the primary analysis to identify potential drug targets. In this analysis, proteins were considered as exposures and PsO as the outcome variable. The protein pQTL and GWAS phenotype data were derived from two sources with no overlap. Only pQTLs that met the following criteria were selected as instrumental variables: (i) pQTLs were cis associations (within 1 MB from the gene encoding the protein) and were used as effective proxies for the proteins. (ii) a significant Bonferroni-corrected genetic association threshold ($1.7\times 10^{-11}$) to control type I errors ($5\times 10^{-8}/n_{protein}$). (iii) SNPs associated with fewer than three proteins to reduce classic horizontal pleiotropy [26]. Finally, we obtained 1954 lead cis-pQTL (lowest *p*-value associations) as proxies for 1954 proteins for ratio estimates to explore potential candidate protein targets, with multiple testing corrections, $p<2.56\times 10^{-5}$ (0.05/1954).

Subsequently, multi-instrument proteome-wide Mendelian randomization analysis employing the IVW method (ratio estimate for the single instrument) was conducted on the explored proteins at the significant threshold ($p<2.56\times 10^{-5}$), excluding dependent associations (linkage disequilibrium (LD) clumping $r^{2}<0.001$) to eliminate pleiotropy. The MR-Egger and weighted median methods were employed to enhance the robustness of the results. Strong cis-pQTL instrumental variables (first stage F-statistic greater than 10, *R*^2^ = 2 × EAF × (1 − EAF) × beta^2^; *F* = *R*^2^ × (*N* − 2)/(1 − *R*^2^)) were used to enhance the strength of the instrumental variables and reduce bias [27]. In both exploratory and confirmatory analyses, GWAS data for PsO used as the outcome, were derived from the FinnGen research project. SMR analysis was performed using the “gsmr” (V1.0.6) R package to ensure the robustness of the results. The instruments for exploratory MR and multi-instrument MR analyses are shown in Supplementary Tables S3 and S4.

MR was performed for reproducibility validation, with a *p*-value threshold of 0.05. Combined pQTL data from the Iceland and EPIC-Norfolk cohorts were used for reproducibility validation. The selection criteria for pQTLs were the same as those for the aforementioned strategy, noting that the significance threshold for pQTLs selection might vary depending on the number of proteins. GWAS phenotype data of PsO from the UK Biobank and GWAS Catalog were used for this validation.

### 2.5. Reverse Causality Detection

To eliminate potential reverse causality, we employed reverse MR-IVW and MR-Egger analyses, adhering to the same criteria for selecting instrumental variables as in the pQTL analysis [28]. This approach aimed to confirm that the causal direction flows from drug-protein targets to disease, rather than the inverse, with a significance threshold set at p<0.05. The GWAS of protein-level data was derived from the UK Biobank.

### 2.6. Bayesian Colocalization Analysis

Bayesian colocalization was employed to assess whether the protein and phenotype share a genetic variant [29], thereby excluding linkage disequilibrium (LD)-mediated horizontal pleiotropy. In this study, we considered using $PPH_{4}>0.9$ as colocalization evidence to support the hypothesis that the protein and PsO share the same genetic variant. The colocalization regions were defined similarly to cis-pQTL regions, as being within 1 MB of the gene that encodes the protein. The parameters used for Bayesian colocalization were $p1=1\times 10^{-4}$ (prior probability: a SNP is associated with protein), $p2=1\times 10^{-4}$ (prior probability: a SNP is associated with PsO), and $p12=1\times 10^{-5}$ (prior probability: a SNP is associated with both protein and PsO). We also set p12 to $5\times 10^{-6}$ ($PPH_{4}>0.8$) as a sensitivity analysis to enhance the robustness of the results. Colocalization analysis was conducted within a 500 kb region upstream and downstream of the gene-coding regions of the identified candidate causal proteins. Colocalization analysis was conducted using the “coloc” (V5.2.3) R package [29], and the results were visualized with the “LocusCompareR” (V1.0.0) R package [30].

### 2.7. Horizontal Pleiotropy Detection

Due to the extensive linkage disequilibrium (LD) present in genetic variations, the observed effect of a protein may be influenced by the effects of other proteins arising from biological pleiotropy [31]. The causal effect observed between the proxy pQTL of a causal protein and the disease may be driven by proxy pQTLs of other proteins that are linked to the pQTLs of the proxy causal protein (Supplementary Figure S1). To mitigate the impact of horizontal pleiotropy arising from this, we performed Mendelian randomization analysis following these steps: First, we identified suspicious genetic variants with an LD $r^{2}$ greater than 0.8 with the proxy pQTL of the causal protein in the cis-region (within 1 MB from the gene encoding the protein). Next, we selected all proteins within the cis-region of the causal protein. Finally, we conducted Mendelian randomization between the pQTLs ($p<5\times 10^{-8}$) of these proteins and PsO. By following these steps, we can exclude the influence of other proteins located within the coding region of the causal protein on its causal effect. The selection of proteins encompassed all proteins from the UK Biobank Pharma Proteomics Project.

### 2.8. Single-Cell RNA-Seq Differential Expression Analysis

To provide additional causal evidence supporting the relationship between plasma proteins and PsO, we performed differential expression analysis of candidate genes by comparing psoriatic and normal skin samples and expression patterns across distinct cell types. Single-cell RNA-seq data were obtained from the Gene Expression Omnibus (GEO) database (GSE183047) [32]. Information on the patients and controls is provided in Supplementary Table S5. Data normalization and quality control were conducted using the “Seurat” (V5.1.0) R package [33], excluding genes with fewer than three cell counts and cells with fewer than 200 unique feature counts. Ultimately, this process yielded 23,245 genes and 18,241 cells across 10 psoriatic skin biopsy samples and 23,245 genes and 13,589 cells across 10 normal skin control samples. We used markers from seven cell types for cell type annotation (Supplementary Table S6). Subsequently, data integration was performed using the Harmony method, and the Wilcoxon rank sum test was conducted to compare the differential expression of candidate genes between psoriatic and normal skin samples, as well as across different cell types. Differential expression: A log fold change greater than 0.5, and a Bonferroni-corrected *p*-value less than 0.05 were considered statistically significant.

### 2.9. Protein-Protein Interaction Networks and Drug Targets Analysis

Protein-protein interaction (PPI) networks were used to explore the interactions between PsO-associated proteins and existing drug targets of approved PsO medications, which were derived from a recent retrospective review [10] that summarized the existing signaling pathways, drugs, and drug targets for PsO (Supplementary Table S7). By analyzing PsO mechanisms, therapeutic targets, and key pathways, we aimed to evaluate the potential of causal proteins as therapeutic targets. All protein-protein interaction analyses were performed using the STRING (V12.0) database [34] (minimum required interaction score of 0.4, with interactions supported by curated databases and experimental evidence given particular emphasis). Detailed information on these drugs was obtained from ChEMBL [35] and DrugBank [36] databases.

### 2.10. Molecular Docking Analysis of Therapeutic Target Proteins

Structure-based virtual screening involves docking small organic molecules with biological and therapeutic targets and is a highly popular computational technique for identifying new chemical scaffolds with biological activities. This technique has yielded many positive results comparable to those of experimental screening [37,38]. The key to successful docking is the use of energy estimation method. The protein structures used as ligands were obtained from the RCSB PDB database [39], and the drug molecule structures used as receptors were sourced from the PubChem database [40]. We used computational molecular docking to assess whether existing PsO drugs could target the identified protein targets and to explore whether drugs not currently used for PsO might interact with these targets, thereby offering a potential for repurposing. Molecular docking was used to evaluate the potential of the identified proteins as therapeutic targets. We downloaded the drug molecular structures from PubChem and the protein structures from the RCSB Protein Data Bank. Molecular docking was performed using AutoDock4, with preprocessing steps including hydrogen addition, water removal, and ligand deletion. A Genetic Algorithm was employed for 50 docking runs, and the results were visualized using PyMOL.

## 3. Results

### 3.1. Proteome Screening for Causal Proteins in PsO

**Figure 2:**
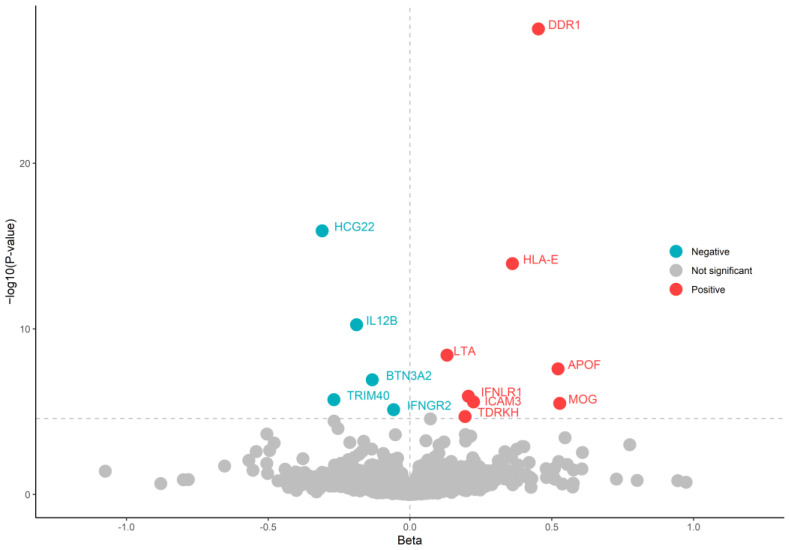
Volcano plot presenting the MR results using lead cis-pQTL.

Following exploratory analysis and confirmation through multi-instrument Mendelian randomization, we identified 13 proteins at Bonferroni significant ($p<2.56\times 10^{-5}$) (Table 1 and Figure 2). No heterogeneity was detected in the Mendelian randomization analyses. The selection of instrumental variables was rigorously conducted according to the aforementioned criteria to ensure the strength of the instruments. High expression levels of eight of these proteins (Interferon lambda receptor 1 (IFNLR1); Apolipoprotein F (APOF); Tudor and KH domain-containing protein (TDRKH); Epithelial discoid in domain-containing receptor 1 (DDR1); HLA class I histocompatibility antigen alpha chain E (HLA-E); Lymphotoxin-alpha (LTA); Myelin-oligodendrocyte glycoprotein (MOG); Intercellular adhesion molecule 3 (ICAM3)) were associated with an increased risk of PsO, while the expression of the remaining five proteins (Interferon gamma receptor 2 (IFNGR2), Protein PBMUCL2 (HCG22); Interleukin-12 subunit beta (IL12B); Butyrophilin subfamily 3 member A2 (BTN3A2); E3 ubiquitin ligase TRIM40 (TRIM40)) was associated with a reduced risk of PsO. All proteins passed the SMR analysis (Supplementary Table S8). The results of exploratory Mendelian randomization analyses using lead cis-pQTL for all proteins are shown in Figure 2 and Supplementary Table S9. The results of the multi-instrument MR analysis of the 13 candidate proteins are shown in Table 1 and Supplementary Table S10.

In the validation analysis, Reverse Mendelian randomization and Bayesian colocalization were employed to exclude interference from other causal pathways and identify the final causal proteins. Firstly, in the reverse Mendelian randomization analysis, only MOG (p=0.038) and BTN3A2 (p=0.044) showed significant results among the 13 proteins (Table 1). However, their MR-Egger results for *p* values were 0.134 and 0.315, respectively, and both did not pass the heterogeneity test. Consequently, MOG and BTN3A2 are not considered to have reverse causal effects (Supplementary Table S11). Subsequent Bayesian colocalization analysis provided strong evidence supporting that IFNLR1 (rs139958347, $PPH_{4}=0.999$), IFNGR2 (rs9808753, $PPH_{4}=0.962$), APOF (rs2020854, $PPH_{4}=0.911$), and TDRKH (rs4845556, $PPH_{4}=0.914$) share the same genetic variants with PsO (Supplementary Table S12). This excluded the influence of LD horizontal pleiotropy and further supported the causal effects. Supplementary Figures S2–S12 present the colocalization results between the proteins and PsO treatment.

Subsequently, we performed horizontal pleiotropy detection for the four causal proteins in PsO. The genetic variations with an LD $r^{2}$ greater than 0.8, and the causal proteins within the cis-region can be found in Supplementary Table S13. The selected genetic variants representing pQTLs did not meet the criteria ($p<5\times 10^{-8}$) or lacked sufficient instrument strength, resulting in no significant Mendelian randomization (MR) results across PsO. This excludes the impact of classical horizontal pleiotropy on the causality.

In the stratified analysis of PsO subtypes (Supplementary Table S14), eight (DDR1, BTN3A2, TRIM40, HLA-E, HCG22, IL12B, LTA, and APOF) of the 13 proteins were associated with the risk of guttate PsO. Three proteins (ICAM3, IL12B, and IFNLR1) were associated with the risk of generalized pustular PsO. Two proteins (DDR1 and TRIM40) were associated with the risk of pustulosis palmaris et plantaris. No heterogeneity was observed in the significant results.

### 3.2. Reproducibility Validation of Potential Drug Targets for PsO

**Figure 3:**
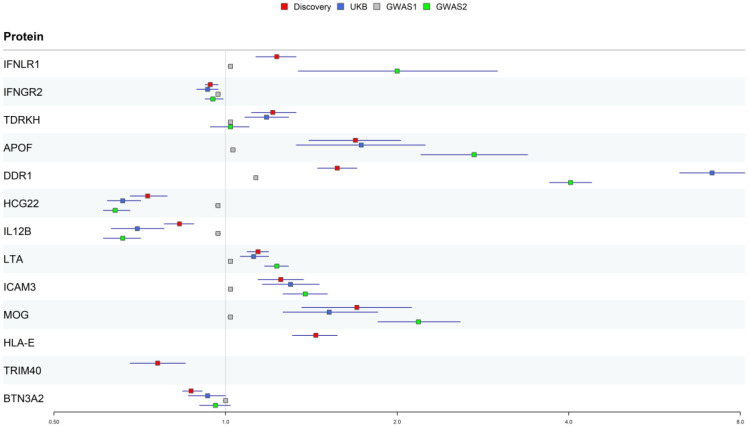
Estimates from the discovery and replication datasets for the 13 identified candidate proteins.

In the reproducibility analysis, meta-proteins from the Icelandic and EPIC-Norfolk cohorts were used. PsO phenotype data were sourced from the Pan-UKB project and two studies available in the GWAS Catalog. We also used the IVW and ratio estimation methods, and ultimately, ten out of the 13 identified proteins demonstrated associations with PsO risk in at least two external datasets (Table 2 and Figure 3). Due to limited data availability, HLA-E and TRIM40 could not be validated in the replication analysis. We classified these proteins into four tiers. Four proteins (IFNLR1, IFNGR2, APOF, and TDRKH) that passed all the aforementioned tests were classified as tier 1. Six proteins (DDR1, HCG22, IL12B, LTA, MOG, and ICAM3) that did not pass the Bayesian colocalization analysis were classified into tier 2. HLA-E and TRIM40, lacking external validation data, were classified as tier 3. BTN3A2 was classified as tier 4 due to the failure to pass any external validations.

### 3.3. Single-Cell Gene Expression Analysis in Psoriatic and Normal Skin

**Figure 4:**
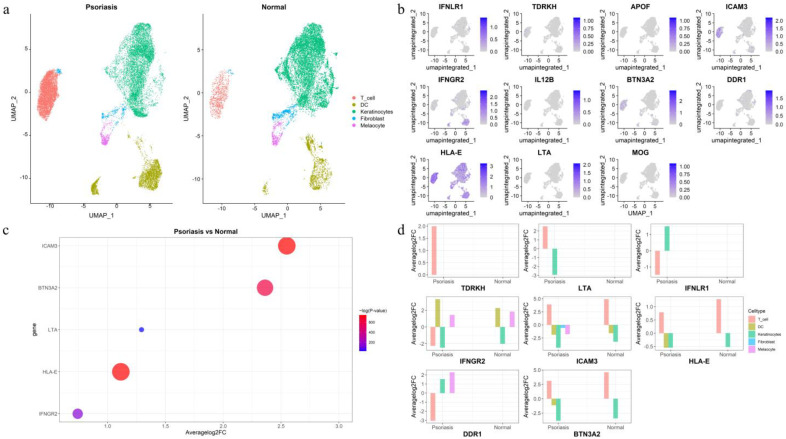
Single-cell expression of the identified candidate protein-coding genes in psoriatic biopsy and normal skin samples. (**a**) Five cell types from psoriatic biopsy and normal skin samples. (**b**) Expression of 11 protein-coding genes in different cell types. (**c**) Differentially expressed genes between psoriatic biopsy and normal skin samples. (**d**) Differential expression of eight protein-coding genes among different cell types in psoriatic and normal skin samples.

To validate the causal effects of the 13 identified protein-coding genes and investigate their cell type specificity, we performed a comprehensive single-cell differential expression analysis between psoriatic and normal skin samples. The cells were clustered into five cell types (T cells, dendritic cells, fibroblasts, keratinocytes, and melanocytes). Notably, the expression level of T cells in psoriatic biopsy samples was higher than that in normal controls (Figure 4a). Expression profiling revealed that 11 of the 13 protein-coding genes were detectable in psoriatic biopsy samples, with HCG22 and TRIM40 being undetectable Figure 4b. In normal skin samples, ten protein-coding genes were expressed, with HCG22, APOF, and TRIM40 remaining undetectable (Supplementary Figure S13). APOF was expressed in psoriatic skin samples but not in normal skin samples, suggesting a potential role in the pathogenesis of psoriasis. In contrast, the absence of HCG22 and TRIM40 expression in the single-cell transcriptomic analysis may be attributed to the characteristics of the sampled tissues and their exclusion from Tier 1 prioritization. Using a log2 fold change (log2FC) greater than 0.5, we identified differential expression of ICAM3, BTN3A2, LTA, HLA-E, and IFNGR2 between psoriatic and normal skin samples (Figure 4c). For cell-type-specific expression, IFNLR1, TDRKH, HLA-E, and DDR1 were predominantly enriched in T cells within psoriatic biopsy samples, a pattern of enrichment not observed in normal skin. IFNGR2 is predominantly enriched in dendritic cells (DC). LTA, ICAM3, and BTN3A2 were mainly enriched in keratinocytes. Notably, in normal skin samples, no statistically significant differences were observed between T cells and other cell types for IFNLR1, IFNGR2, LTA, TDRKH, and DDR1 expression (Figure 4d). However, in patients with PsO, these genes exhibited specific expression in T cells.

### 3.4. Protein-Protein Interaction Analysis Between Candidate Therapeutic Targets and Current PsO Medications

**Figure 5:**
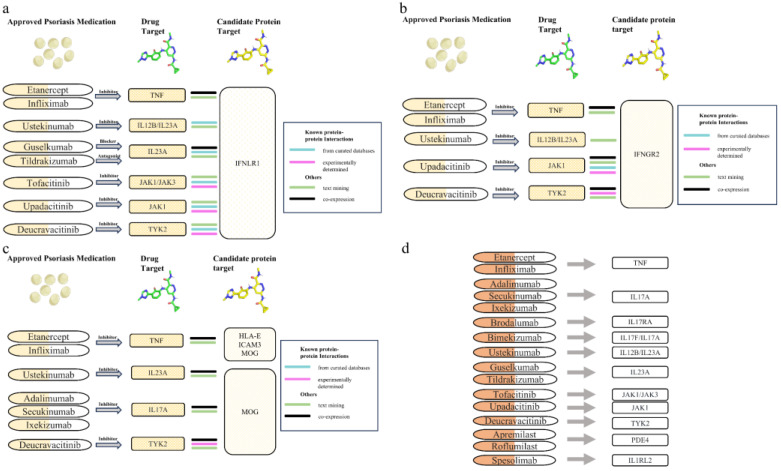
Protein-protein interaction (PPI) analysis was used to show interactions between PsO drug targets and identified potential drug targets. Figures (**a**–**c**) illustrate the interactions between PsO drug targets and IFNLR1, IFNGR2, HLA-E, ICAM3, and MOG. Figure (**d**) shows the current PsO drug targets and their associated drugs.

PPI analysis revealed interactions between two tier 1 proteins (IFNLR1 and IFNGR2) and three tier 2 proteins (IL12B, LTA, MOG, and ICAM3) with existing drug targets (Figure 5). Among them, IL12B and LTA are already established therapeutic targets for PsO, and IFNGR2 has been developed as a therapeutic target for chronic granulomatous disease. High-confidence interactions were identified through PPI network analysis for the following pairs (curated databases and experimental evidence): IFNLR1-TYK2, IFNLR1-JAK1, IFNLR1-JAK3, IFNLR1-IL23A, IFNLR1-IL12B, IFNGR2-TYK2, IFNGR2-JAK1 and MOG-TYK2. In particular, the non-receptor tyrosine-protein kinase TYK2 (TYK2, the target of deucravacitinib) and tyrosine-protein kinase JAK1 (JAK1, the target of upadacitinib) are known to have established connections with both IFNLR1 and IFNGR2. These interactions have been confirmed through physical experiments and are supported by multiple databases. Additionally, PPI analysis revealed high-confidence interactions between IFNLR1 and tyrosine-protein kinase JAK3 (JAK3, the target of tofacitinib), interleukin-23 subunit alpha (IL23A, the target of guselkumab and tildrakizumab), and interleukin-12 subunit beta (IL12B, the target of ustekinumab). IFNLR1 and ICAM3 are associated with tumor necrosis factor (TNF, a target of etanercept and infliximab), indicating that they may be involved in shared cellular processes. IFNGR2 is also linked to TNF and IL12B. MOG interacts with multiple PsO drug targets, such as Interleukin-17A (IL-17A, the target of secukinumab), TYK2, and IL-23A.

### 3.5. Molecular Docking Analysis for Druggability Evaluation

**Figure 6:**
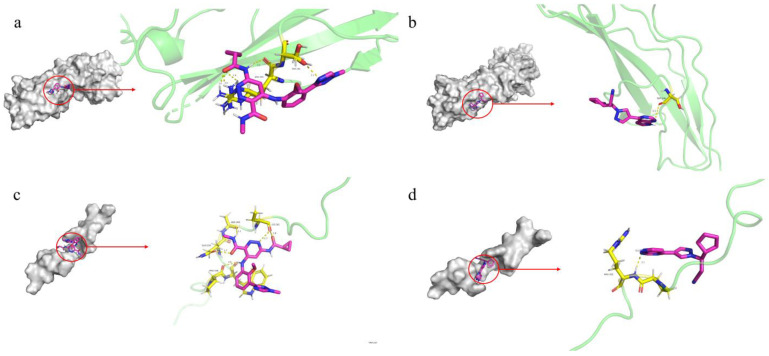
Molecular docking pattern of IFNLR1 and IFNGR2 with two PsO medications. (**a**) IFNGR2-Deucravacitinib (binding energy = −6.61 kcal/mol). (**b**) IFNGR2-Ruxolitinib (binding energy = −6.43 kcal/mol). (**c**) IFNLR1-Deucravacitinib (binding energy = −5.24 kcal/mol). (**d**) IFNLR1-Ruxolitinib (binding energies = −6.05 kcal/mol).

The JAK-STAT and TYK2 pathways play critical roles in cytokine signaling in PsO and are strongly associated with the candidate target proteins. We searched the DrugBank database for drugs targeting TYK2 and JAK1, ultimately selecting the PsO medication deucravacitinib (an inhibitor of TYK2) and the anticancer drug ruxolitinib (an inhibitor of JAK1 and JAK2) as ligands. We then performed molecular docking using IFNLR1 and IFNGR2 as receptors. The binding energies of IFNLR1-Deucravacitinib (−5.24 kcal/mol), IFNLR1-Ruxolitinib (−6.05 kcal/mol), IFNGR2-Deucravacitinib (−6.61 kcal/mol), and IFNGR2-Ruxolitinib (−6.43 kcal/mol) were all less than −5 kcal/mol (Figure 6 and Supplementary Table S15), indicating that both drugs have the potential to form stable interactions with the potential causal proteins IFNLR1 and IFNGR2, suggesting their therapeutic potential for treating PsO.

## 4. Discussion

To our knowledge, this is the largest study to date exploring PsO targets using plasma proteins, with the most rigorous replication (three independent datasets), and integrates single-cell data to examine the expression patterns of causal proteins and assess their druggability. In this study, we identified 13 proteins as potential therapeutic targets for PsO through a proteome-wide Mendelian randomization analysis of 1954 plasma proteins. Ten of the 13 identified proteins were associated with the risk of PsO subtypes (Guttate PsO, Pustulosis palmaris et plantaris, and generalized pustular PsO). By integrating reverse Mendelian randomization, SMR analysis, and horizontal pleiotropy assessment, we established a causal association between protein and disease. Bayesian colocalization indicated that four proteins shared the same variants with PsO, supporting their causal association. Repeated MR validated 10 of the 13 proteins across at least two datasets [41]. We further utilized single-cell data to validate the expression differences of the encoding protein-coding genes. In this study, rigorous causal validation for IFNLR1 and IFNGR2, along with our novel finding of their specific expression in T cells from PsO biopsy samples and the promising drug evaluation results revealed through molecular docking, collectively highlight their therapeutic potential. Furthermore, a study by Lazear et al. suggested that the IFN family may represent a promising direction for psoriasis treatment [42], while our study revealed the IFN receptor proteins IFNLR1 and IFNGR2, highlighting their therapeutic potential for PsO. Meanwhile, LTA, TDRKH, and DDR1 were expressed exclusively in T cells from psoriasis biopsy samples, while HLA-E and ICAM3 exhibited psoriasis-specific- expression. These findings offer new perspectives for mechanistic psoriasis research.

IFNLR1 and interleukin 10 receptor subunit beta (IL-10RB) form a unique dimer known as the IFN-λ (type III IFN) receptor, which serves as a receptor for the cytokines IFNL1, IFNL2, IFNL3, and IFNL4 [43,44]. The binding of IFN-λ to its receptor activates the JAK-STAT pathway, which plays a crucial role in inflammatory and immune diseases. Moreover, IFN-λ plays a role in autoimmune diseases and inflammatory responses [45]. In our PPI analysis, we found that IFNLR1 is linked to several existing drug targets, including IL12B/IL23A, JAK, and TYK2, with known interactions. Previous studies have indicated that IL12/IL23 and IL23 inhibitors can provide prolonged efficacy even after discontinuation [46], while JAK inhibitors, which have been recently approved, show promising safety profiles and are currently undergoing clinical trials, making them a highly promising class of drugs [10]. Numerous studies have demonstrated that type III IFN plays a pivotal role in immune responses at barrier surfaces [42,47,48]. Chemokines produced by IFN-λ act on keratinocytes and epithelial cells, inducing inflammatory responses and attracting immune cells. Research on IFN-λ gene polymorphisms has also revealed that G alleles may be closely linked to the pathophysiological processes of PsO [49]. As a receptor for type III interferons, IFNLR1 has the potential to serve as a drug target for PsO. Additionally, the favorable molecular docking results of INFLR1 with the JAK1 inhibitor ruxolitinib (an anticancer drug) and the TYK2 inhibitor deucravacitinib (a PsO medication) (<−5 kcal/mol) also suggest that IFNLR1 could be a viable drug target and that existing non-PsO medications might be repurposed for PsO treatment.

Compared to IFNLR1, IFNGR2, and IFNGR1 form the interferon-gamma receptor complex, which, upon binding to the IFN-γ (type II IFN) ligand, similarly activates the JAK-STAT pathway [50]. Activation of the JAK-STAT1 pathway further induces the expression of the pro-inflammatory cytokine TNF-α, an established PsO target [51]. The development or exacerbation of PsO may be triggered or worsened by various cellular changes induced by IFN-γ, such as the abnormal differentiation of keratinocytes [52] and protein-mediated responses [53]. Type II IFN may influence PsO by modulating pathways related to complement, proteases, and upstream miRNAs [54]. As a crucial receptor for IFN-γ, IFNGR2 may serve as a potential target for influencing the progression of PsO. Moreover, additional research has revealed the role of the IFN family in autoimmune diseases [55,56]. Currently, there are drugs that target IFNGR2. Actimmune, which binds to IFNGR2, is known to alleviate severe malignant osteoporosis and chronic granulomatous disease and may potentially be repurposed for PsO.

A previous MR analysis by Cai et al. [57] identified a role for IFNLR1 in psoriatic arthritis, which is consistent with our finding of IFNLR1 as a therapeutic target for psoriasis, collectively suggesting its involvement in psoriasis progression. In addition, IFNGR2 was identified in our study but has not been reported in prior research, indicating that the IFN family warrants further attention and plays a role in the broader spectrum of PsO. Moreover, our study revealed specific expression of IFNLR1 and IFNGR2 in T cells from psoriatic skin biopsy samples compared to normal skin, a pattern not observed by Cai et al. in the peripheral blood samples of psoriasis patients. Furthermore, our molecular docking results also demonstrated their potential as drug targets.

APOF is a liver-synthesized and secreted protein. Audrey et al. provided new insights into APOF, indicating that increased APOF levels reduce plasma triglyceride levels and elevate high-density lipoprotein (HDL) levels, thereby exerting protective effects against cardiovascular diseases [58]. Interestingly, recent studies have revealed the pleiotropic effects of PCSK9 inhibitors—a cardiovascular drug that regulates low-density lipoprotein cholesterol—on autoimmune diseases [59], as well as the impact of APOF on type 2 diabetes. These findings suggest that APOF and cardiovascular drugs may play potential roles in autoimmune diseases. APOF, a promising target, was also mentioned in the MR study by Liu et al. [60], who suggested that the association between APOF and psoriasis may be mediated through its effects on atherosclerosis. The functional relationship between APOF and psoriasis requires further experimental investigation. Tudor and TDRKH are proteins essential for spermatogenesis and are involved in the biogenesis of piRNA [61]. Despite our study suggesting a potential causal relationship between TDRKH and PsO, there is currently no research linking TDRKH to autoimmune diseases. Further investigation is needed to elucidate the role of TDRKH in non-reproductive processes.

Additionally, tier 2 and tier 3 candidate causal proteins are implicated in the progression of autoimmune or inflammatory diseases. DDR1 inhibits the host’s anti-tumor immunity, and anti-DDR1 antibodies could serve as an alternative anticancer immunotherapy for a variety of cancer types [62]. A subset of patients with antibodies against MOG expresses a clinical phenotype distinct from multiple sclerosis (MS) [63]. Moreover, MOG drives Th17/Th1 cells to produce IL-17A and TNF-α, which may have a potential impact on psoriasis [64]. ICAM3 is involved in immune cell interactions and T-lymphocyte activation, influencing inflammatory pathways [65]. Studies have also shown that ICAM1, which shares similar functions with ICAM3, may be induced by TNF-α, thereby enhancing neutrophil migration to the sites of inflammation [66]. TRIM40 may serve as a therapeutic target to limit the onset and progression of inflammatory bowel disease (IBD) [67]. These are potential drug targets for PsO that warrant further investigation.

The strength of this study lies in its rigorous exclusion of alternative causal pathways using only cis-pQTLs in both the discovery and external validation cohorts. This approach ensured the establishment of a causal relationship between candidate causal proteins and PsO. Compared to other similar studies, we employed horizontal pleiotropy detection to rule out the possibility that other proteins mediated the effects of the identified proteins. Single-cell differential expression analysis further validated the causal effects of protein-coding genes on PsO, revealing distinct expression patterns across various cell types. The accurate identification of approved drug targets (IL12B and LTA) for PsO in this study also validated the reliability of the research. They are also likely to be developed as therapies for guttate PsO and pustular PsO. Additionally, these identified proteins (e.g., DDR1, APOF, and TRIM40) associated with PsO subtypes are also worthy of attention. The large-scale plasma protein data used in our study from the UK Biobank ensured the homogeneity and robustness of the research. Furthermore, the results of the PPI analysis and molecular docking provide supporting evidence for the potential of these candidate proteins to serve as drug targets for PsO. The application of drug molecular docking offers a novel perspective for the potential repurposing of existing medications; however, these results reflect only computationally simulated binding affinities and should be interpreted with caution. The identified proteins, IFNLR1 and IFNGR2, underscore the significance of IFNs in the context of PsO. Their associations with multiple PsO drug targets, roles in disease progression, and potential for drug development highlight their potential as new therapeutic targets for PsO. Nevertheless, this study has several limitations. Firstly, our study primarily focused on the European population, necessitating further investigation into non-European ancestries. Secondly, although we only utilized pQTL data for analysis, stringent significance thresholds may have filtered out some potentially critical proteins involved in the pathogenesis of PsO. Additionally, further functional validation experiments are required to confirm the roles of these identified proteins. Third, due to the lack of available GWAS data, we were unable to replicate the associations for HLA-E, TRIM40, and BTN3A2 also failed to replicate. This represents a limitation of the present study. More comprehensive proteomic and GWAS datasets, as well as additional related studies, are needed to address this gap.

## 5. Conclusions

In summary, our study reveals a causal relationship between several plasma proteins and PsO. Notably, IFNLR1 and IFNGR2 are highly likely to serve as candidate therapeutic targets for PsO and provide etiological support for the role of IFNs in the pathogenesis of the disease, warranting further experimental and clinical studies to elucidate their mechanisms of action.

## Figures and Tables

**Table 1:** Summary of causal validation results for bidirectional MR, reproducibility, and colocalization of 13 candidate proteins.

Protein	UniProt	*P_discovery_*	*P_replication_* ^a^	*P_reverse_*	Colocalization ^b^ $PPH_{4}$	Category
IFNLR1	Q8IU57	1.17e−06	4.49e−04	0.221	0.999/0.998	Tier1
IFNGR2	P38484	7.54e−06	4.77e−04	0.640	0.962/0.929	Tier1
TDRKH	Q9Y2W6	1.94e−05	7.11e−06	0.349	0.915/0.846	Tier1
APOF	Q13790	2.56e−08	6.20e−20	0.864	0.911/0.836	Tier1
DDR1	Q08345	7.73e−29	2.05e−228	0.082	0.000/0.000	Tier2
HCG22	E2RYF7	1.19e−16	2.51e−56	0.117	0.000/0.000	Tier2
IL12B	P29460	5.56e−11	1.20e−23	0.729	0.000/0.000	Tier2
LTA	P01374	2.54e−09	2.81e−15	0.063	0.000/0.000	Tier2
ICAM3	P32942	2.54e−06	2.56e−12	0.895	0.034/0.017	Tier2
MOG	Q16653	3.14e−06	2.74e−20	0.038	0.000/0.000	Tier2
HLA-E	P13747	1.14e−14	NA	0.354	0.000/0.000	Tier3
TRIM40	Q6P9F5	1.87e−06	NA	0.209	0.000/0.000	Tier3
BTN3A2	P78410	2.46e−09	5.09e−02	0.044	0.000/0.000	Tier4

^a^ The reproducibility validation results were conducted using the UKB and two GWAS Catalog datasets, but only the most significant *p*-values were presented. ^b^ prior probability p12 (1e−5/5e−6).

**Table 2:** MR results from the discovery set and external reproducibility validation for the 13 candidate proteins.

	Discovery		UKB		GWAS1		GWAS2	
Protein	OR (95%CI)	*p*	OR (95%CI)	*p*	OR (95%CI)	*p*	OR (95%CI)	*p*
IFNLR1	1.23 (1.13–1.33)	1.17e−06	NA	NA	1.01 (1.01–1.02)	4.49e−04	2 (1.34–3)	7.25e−04
IFNGR2	0.94 (0.92–0.97)	7.54e−06	0.93 (0.89–0.97)	4.77e−04	1 (0.99–1)	8.72e−04	0.95 (0.92–0.99)	4.88e−03
TDRKH	1.21 (1.11–1.33)	1.94e−05	1.18 (1.08–1.29)	1.51e−04	1.02 (1.01–1.02)	7.11e−06	1.02 (0.94–1.1)	6.86e−01
APOF	1.69 (1.4–2.03)	2.56e−08	1.73 (1.33–2.24)	4.19e−05	1.03 (1.02–1.04)	2.80e−06	2.73 (2.2–3.39)	6.20e−20
DDR1	1.57 (1.45–1.7)	7.73e−29	7.14 (6.26–8.15)	3.11e−186	1.13 (1.12–1.14)	2.04e−199	4.03 (3.7–4.39)	2.05e−228
HCG22	0.73 (0.68–0.79)	1.19e−16	0.66 (0.62–0.71)	1.12e−30	0.97 (0.96–0.97)	1.34e−30	0.64 (0.61–0.68)	2.51e−56
IL12B	0.83 (0.78–0.88)	5.56e−11	0.7 (0.63–0.78)	3.69e−11	0.97 (0.96–0.98)	1.62e−12	0.66 (0.61–0.71)	1.20e−23
LTA	1.14 (1.09–1.19)	2.54e−09	1.12 (1.06–1.19)	1.51e−04	1 (1–1.01)	2.34e−03	1.23 (1.17–1.29)	2.81e−15
ICAM3	1.25 (1.14–1.37)	2.54e−06	1.3 (1.16–1.46)	1.21e−05	1.02 (1.02–1.03)	1.83e−07	1.38 (1.26–1.51)	2.56e−12
MOG	1.7 (1.36–2.12)	3.14e−06	1.52 (1.26–1.85)	1.69e−05	1.02 (1.01–1.02)	6.67e−06	2.18 (1.85–2.58)	2.74e−20
HLA-E	1.44 (1.31–1.57)	1.14e−14	NA	NA	NA	NA	NA	NA
TRIM40	0.76 (0.68–0.85)	1.87e−06	NA	NA	NA	NA	NA	NA
BTN3A2	0.87 (0.84–0.91)	2.46e−09	0.93 (0.86–1)	5.09e−02	1 (0.99–1)	5.74e−01	0.96 (0.9–1.02)	2.28e−01

The significance threshold for the discovery set was defined as $p<2.56\times 10^{-5}$, while the threshold for external reproducibility validation was set at p<0.05

## Data Availability

Informed consent was obtained from all subjects involved in the study. The results of this study are available in the article and Supplementary Materials. The quality control of pQTL data and GWAS phenotype data, as well as specific disease information, can be obtained from the following sources. Summary data for the GWAS from the Pan-UK Biobank based on the UK Biobank (project ID 31063) can be accessed at https://pan.ukbb.broadinstitute.org/downloads/index.html (all web accessed on 15 December 2024) [22]. The summary data for the GWAS from FinnGen can be obtained at https://finngen.gitbook.io/documentation/r10 [21]. Summary data for PsO GWAS, GCST90014456 [24], and GCST90019016 [25] from The NHGRI-EBI Catalog can be downloaded at https://www.ebi.ac.uk/gwas/home [23]. The summary data for the GWAS of plasma proteins from the UK Biobank can be found at https://metabolomips.org/ukbbpgwas/ [18]. The summary data for plasma proteins from Iceland is available at https://www.decode.com/summarydata [19]. The summary data for plasma proteins from the EPIC-Norfolk cohort can be accessed at https://www.nature.com/articles/s42255-023-00753-7 [20]. Single-cell RNA-seq data from psoriatic biopsy samples and normal skin were obtained from the Gene Expression Omnibus (GEO), https://www.ncbi.nlm.nih.gov/geo/query/acc.cgi?acc=GSE183047 [32].

## Supplementary Materials

The following supporting information can be downloaded at: https://www.mdpi.com/article/10.3390/biomedicines13061380/s1, Supplementary Table S1: Plasma protein data of this study; Supplementary Table S2: Psoriasis GWAS phenotypic data of this study; Supplementary Table S3: Lead cis-pQTL instruments of plasma proteins for exploratory MR analysis; Supplementary Table S4: Muti cis-pQTL instruments of explored proteins for validation MR analysis; Supplementary Table S5: Information on patients and controls; Supplementary Table S6: Cell annotation marker gene; Supplementary Table S7: Cell annotation marker gene; Supplementary Table S8: SMR analysis for identifed proteins; Supplementary Table S9: Exploratory MR analysis on plasma proteins with lead cis-pQTL instrument; Supplementary Table S10: Validation MR analysis on plasma proteins with muti cis-pQTL instruments; Supplementary Table S11: Reverse MR analysis for psoriasis on levels of ten candidate proteins; Supplementary Table S12: Bayesian colocalization analysis of ten potential causal proteins and psoriasis; Supplementary Table S13: Proxy snp, snps associated with proxy snp and proteins in cis-region of four candidate causal proteins; Supplementary Table S14: Mendelian randomization analysis for psoriasis subtypes; Supplementary Table S15: Molecular docking of IFNLR1 and IFNGR2 with psoriasis drugs. Figure S1: Horizontal pleiotropy may result in false causality, leading to ineffective drug targets; Supplementary Figure S2: The association plot of the colocalization region between APOF and psoriasis; Supplementary Figure S3: The association plot of the colocalization region between DDR1 and psoriasis; Supplementary Figure S4: The association plot of the colocalization region between HCG22 and psoriasis; Supplementary Figure S5: The association plot of the colocalization region between ICAM3 and psoriasis; Supplementary Figure S6: The association plot of the colocalization region between IFNGR2 and psoriasis; Supplementary Figure S7: The association plot of the colocalization region between IFNLR1 and psoriasis; Supplementary Figure S8: The association plot of the colocalization region between IL12B and psoriasis; Supplementary Figure S9: The association plot of the colocalization region between MOG and psoriasis; Supplementary Figure S10: The association plot of the colocalization region between TDRKH and psoriasis; Supplementary Figure S11: The association plot of the colocalization region between BTN3A2 and psoriasis; Supplementary Figure S12: The association plot of the colocalization region between LTA and psoriasis; Supplementary Figure S13: Single-cell expression of protein-coding genes in normal skin samples.

## Abbreviations

MR, Mendelian randomization; pQTL, protein quantitative trait loci; SMR, Summary-data-based Mendelian randomization; PPI, Protein-protein interaction; WHO, World Health Organization; SNPs, single nucleotide polymorphisms; GWAS, genome-wide association studies; IVW, Inverse-variance weighted; GEO, Gene Expression Omnibus; log2FC, log2 fold change; DC, Dendritic cells; MOG, Myelin oligodendrocyte glycoprotein; MS, Multiple sclerosis; IBD, Inflammatory bowel disease.

## References

[1] Griffiths C.E., Barker J.N. (2007). Pathogenesis and Clinical Features of Psoriasis. Lancet.

[2] Christophers E. (2001). Psoriasis—Epidemiology and Clinical Spectrum. Clin. Exp. Dermatol..

[3] Parisi R., Iskandar I.Y.K., Kontopantelis E., Augustin M., Griffiths C.E.M., Ashcroft D.M. (2020). National, Regional, and Worldwide Epidemiology of Psoriasis: Systematic Analysis and Modelling Study. BMJ.

[4] Lee J.H., Kim H.J., Han K.D., Kim H.-N., Park Y.M., Lee J.Y., Park Y.-G., Lee Y.B. (2019). Cancer Risk in 892 089 Patients with Psoriasis in Korea: A Nationwide Population-Based Cohort Study. J. Dermatol..

[5] Egeberg A., Thyssen J.P., Gislason G.H., Skov L. (2016). Skin Cancer in Patients with Psoriasis. J. Eur. Acad. Dermatol. Venereol..

[6] Dai H., Li W.-Q., Qureshi A.A., Han J. (2016). Personal History of Psoriasis and Risk of Nonmelanoma Skin Cancer (NMSC) among Women in the United States: A Population-Based Cohort Study. J. Am. Acad. Dermatol..

[7] Vaengebjerg S., Skov L., Egeberg A., Loft N.D. (2020). Prevalence, Incidence, and Risk of Cancer in Patients with Psoriasis and Psoriatic Arthritis. JAMA Dermatol..

[8] Griffiths C.E.M., Armstrong A.W., Gudjonsson J.E., Barker J.N.W.N. (2021). Psoriasis. Lancet.

[9] Svoboda S.A., Ghamrawi R.I., Owusu D.A., Feldman S.R. (2020). Treatment Goals in Psoriasis: Which Outcomes Matter Most?. Am. J. Clin. Dermatol..

[10] Guo J., Zhang H., Lin W., Lu L., Su J., Chen X. (2023). Signaling Pathways and Targeted Therapies for Psoriasis. Signal Transduct. Target. Ther..

[11] Iskandar I.Y.K., Warren R.B., Lunt M., Mason K.J., Evans I., McElhone K., Smith C.H., Reynolds N.J., Ashcroft D.M., Griffiths C.E.M. (2018). Differential Drug Survival of Second-Line Biologic Therapies in Patients with Psoriasis: Observational Cohort Study from the British Association of Dermatologists Biologic Interventions Register (BADBIR). J. Investig. Dermatol..

[12] Warren R.B., Smith C.H., Yiu Z.Z.N., Ashcroft D.M., Barker J.N.W.N., Burden A.D., Lunt M., McElhone K., Ormerod A.D., Owen C.M. (2015). Differential Drug Survival of Biologic Therapies for the Treatment of Psoriasis: A Prospective Observational Cohort Study from the British Association of Dermatologists Biologic Interventions Register (BADBIR). J. Investig. Dermatol..

[13] Zheng J., Haberland V., Baird D., Walker V., Haycock P.C., Hurle M.R., Gutteridge A., Erola P., Liu Y., Luo S. (2020). Phenome-Wide Mendelian Randomization Mapping the Influence of the Plasma Proteome on Complex Diseases. Nat. Genet..

[14] Sanderson E., Glymour M.M., Holmes M.V., Kang H., Morrison J., Munafò M.R., Palmer T., Schooling C.M., Wallace C., Zhao Q. (2022). Mendelian Randomization. Nat. Rev. Methods Primers.

[15] Reay W.R., Cairns M.J. (2021). Advancing the Use of Genome-Wide Association Studies for Drug Repurposing. Nat. Rev. Genet..

[16] King E.A., Davis J.W., Degner J.F. (2019). Are Drug Targets with Genetic Support Twice as Likely to Be Approved? Revised Estimates of the Impact of Genetic Support for Drug Mechanisms on the Probability of Drug Approval. PLoS Genet..

[17] Skrivankova V.W., Richmond R.C., Woolf B.A.R., Yarmolinsky J., Davies N.M., Swanson S.A., VanderWeele T.J., Higgins J.P.T., Timpson N.J., Dimou N. (2021). Strengthening the Reporting of Observational Studies in Epidemiology Using Mendelian Randomization: The STROBE-MR Statement. JAMA.

[18] Sun B.B., Chiou J., Traylor M., Benner C., Hsu Y.-H., Richardson T.G., Surendran P., Mahajan A., Robins C., Vasquez-Grinnell S.G. (2023). Plasma Proteomic Associations with Genetics and Health in the UK Biobank. Nature.

[19] Ferkingstad E., Sulem P., Atlason B.A., Sveinbjornsson G., Magnusson M.I., Styrmisdottir E.L., Gunnarsdottir K., Helgason A., Oddsson A., Halldorsson B.V. (2021). Large-Scale Integration of the Plasma Proteome with Genetics and Disease. Nat. Genet..

[20] Koprulu M., Carrasco-Zanini J., Wheeler E., Lockhart S., Kerrison N.D., Wareham N.J., Pietzner M., Langenberg C. (2023). Proteogenomic Links to Human Metabolic Diseases. Nat. Metab..

[21] Kurki M.I., Karjalainen J., Palta P., Sipilä T.P., Kristiansson K., Donner K.M., Reeve M.P., Laivuori H., Aavikko M., Kaunisto M.A. (2023). Author Correction: FinnGen Provides Genetic Insights from a Well-Phenotyped Isolated Population. Nature.

[22] Karczewski K.J., Gupta R., Kanai M., Lu W., Tsuo K., Wang Y., Walters R.K., Turley P., Callier S., Baya N. (2024). Pan-UK Biobank GWAS Improves Discovery, Analysis of Genetic Architecture, and Resolution into Ancestry-Enriched Effects. medRxiv.

[23] Sollis E., Mosaku A., Abid A., Buniello A., Cerezo M., Gil L., Groza T., Güneş O., Hall P., Hayhurst J. (2023). The NHGRI-EBI GWAS Catalog: Knowledgebase and Deposition Resource. Nucleic Acids Res..

[24] Glanville K.P., Coleman J.R.I., O’Reilly P.F., Galloway J., Lewis C.M. (2021). Investigating Pleiotropy Between Depression and Autoimmune Diseases Using the UK Biobank. Biol. Psychiatry Glob. Open Sci..

[25] Stuart P.E., Tsoi L.C., Nair R.P., Ghosh M., Kabra M., Shaiq P.A., Raja G.K., Qamar R., Thelma B.K., Patrick M.T. (2022). Transethnic Analysis of Psoriasis Susceptibility in South Asians and Europeans Enhances Fine-Mapping in the MHC and Genomewide. HGG Adv..

[26] Davey Smith G., Hemani G. (2014). Mendelian Randomization: Genetic Anchors for Causal Inference in Epidemiological Studies. Hum. Mol. Genet..

[27] Palmer T.M., Lawlor D.A., Harbord R.M., Sheehan N.A., Tobias J.H., Timpson N.J., Smith G.D., Sterne J.A. (2012). Using Multiple Genetic Variants as Instrumental Variables for Modifiable Risk Factors. Stat. Methods Med. Res..

[28] Zhou W., Liu G., Hung R.J., Haycock P.C., Aldrich M.C., Andrew A.S., Arnold S.M., Bickeböller H., Bojesen S.E., Brennan P. (2021). Causal Relationships between Body Mass Index, Smoking and Lung Cancer: Univariable and Multivariable Mendelian Randomization. Int. J. Cancer.

[29] Giambartolomei C., Vukcevic D., Schadt E.E., Franke L., Hingorani A.D., Wallace C., Plagnol V. (2014). Bayesian Test for Colocalisation between Pairs of Genetic Association Studies Using Summary Statistics. PLoS Genet..

[30] Liu B., Gloudemans M.J., Rao A.S., Ingelsson E., Montgomery S.B. (2019). Abundant Associations with Gene Expression Complicate GWAS Follow-Up. Nat. Genet..

[31] Solovieff N., Cotsapas C., Lee P.H., Purcell S.M., Smoller J.W. (2013). Pleiotropy in Complex Traits: Challenges and Strategies. Nat. Rev. Genet..

[32] Kim J., Lee J., Li X., Kunjravia N., Rambhia D., Cueto I., Kim K., Chaparala V., Ko Y., Garcet S. (2023). Multi-Omics Segregate Different Transcriptomic Impacts of Anti-IL-17A Blockade on Type 17 T-Cells and Regulatory Immune Cells in Psoriasis Skin. Front. Immunol..

[33] Hao Y., Stuart T., Kowalski M.H., Choudhary S., Hoffman P., Hartman A., Srivastava A., Molla G., Madad S., Fernandez-Granda C. (2024). Dictionary Learning for Integrative, Multimodal and Scalable Single-Cell Analysis. Nat. Biotechnol..

[34] Szklarczyk D., Gable A.L., Lyon D., Junge A., Wyder S., Huerta-Cepas J., Simonovic M., Doncheva N.T., Morris J.H., Bork P. (2019). STRING V11: Protein–Protein Association Networks with Increased Coverage, Supporting Functional Discovery in Genome-Wide Experimental Datasets. Nucleic Acids Res..

[35] Mendez D., Gaulton A., Bento A.P., Chambers J., De Veij M., Félix E., Magariños M.P., Mosquera J.F., Mutowo P., Nowotka M. (2019). ChEMBL: Towards Direct Deposition of Bioassay Data. Nucleic Acids Res..

[36] Wishart D.S., Feunang Y.D., Guo A.C., Lo E.J., Marcu A., Grant J.R., Sajed T., Johnson D., Li C., Sayeeda Z. (2018). DrugBank 5.0: A Major Update to the DrugBank Database for 2018. Nucleic Acids Res..

[37] Lyu J., Wang S., Balius T.E., Singh I., Levit A., Moroz Y.S., O’Meara M.J., Che T., Algaa E., Tolmachova K. (2019). Ultra-Large Library Docking for Discovering New Chemotypes. Nature.

[38] Kincaid V.A., London N., Wangkanont K., Wesener D.A., Marcus S.A., Héroux A., Nedyalkova L., Talaat A.M., Forest K.T., Shoichet B.K. (2015). Virtual Screening for UDP-Galactopyranose Mutase Ligands Identifies a New Class of Antimycobacterial Agents. ACS Chem. Biol..

[39] Burley S.K., Bhikadiya C., Bi C., Bittrich S., Chao H., Chen L., Craig P.A., Crichlow G.V., Dalenberg K., Duarte J.M. (2023). RCSB Protein Data Bank (RCSB.Org): Delivery of Experimentally-Determined PDB Structures alongside One Million Computed Structure Models of Proteins from Artificial Intelligence/Machine Learning. Nucleic Acids Res..

[40] Kim S., Chen J., Cheng T., Gindulyte A., He J., He S., Li Q., Shoemaker B.A., Thiessen P.A., Yu B. (2023). PubChem 2023 Update. Nucleic Acids Res..

[41] Nelson M.R., Tipney H., Painter J.L., Shen J., Nicoletti P., Shen Y., Floratos A., Sham P.C., Li M.J., Wang J. (2015). The Support of Human Genetic Evidence for Approved Drug Indications. Nat. Genet..

[42] Lazear H.M., Schoggins J.W., Diamond M.S. (2019). Shared and Distinct Functions of Type I and Type III Interferons. Immunity.

[43] Kotenko S.V., Gallagher G., Baurin V.V., Lewis-Antes A., Shen M., Shah N.K., Langer J.A., Sheikh F., Dickensheets H., Donnelly R.P. (2003). IFN-Lambdas Mediate Antiviral Protection through a Distinct Class II Cytokine Receptor Complex. Nat. Immunol..

[44] Henden A.S., Koyama M., Robb R.J., Forero A., Kuns R.D., Chang K., Ensbey K.S., Varelias A., Kazakoff S.H., Waddell N. (2021). IFN-λ Therapy Prevents Severe Gastrointestinal Graft-versus-Host Disease. Blood.

[45] Vlachiotis S., Andreakos E. (2019). Lambda Interferons in Immunity and Autoimmunity. J. Autoimmun..

[46] Masson Regnault M., Shourick J., Jendoubi F., Tauber M., Paul C. (2022). Time to Relapse After Discontinuing Systemic Treatment for Psoriasis: A Systematic Review. Am. J. Clin. Dermatol..

[47] Kotenko S.V., Durbin J.E. (2017). Contribution of Type III Interferons to Antiviral Immunity: Location, Location, Location. J. Biol. Chem..

[48] Lazear H.M., Nice T.J., Diamond M.S. (2015). Interferon-λ: Immune Functions at Barrier Surfaces and Beyond. Immunity.

[49] Yilmaz B., Çakmak Genç G., Karakaş Çelik S., Solak Tekin N., Can M., Dursun A. (2022). Association between Psoriasis Disease and IFN-λ Gene Polymorphisms. Immunol. Investig..

[50] Rosenzweig S.D., Schwartz O.M., Brown M.R., Leto T.L., Holland S.M. (2004). Characterization of a Dipeptide Motif Regulating IFN-Gamma Receptor 2 Plasma Membrane Accumulation and IFN-Gamma Responsiveness. J. Immunol..

[51] Wilcock D.M. (2012). Neuroinflammation in the Aging Down Syndrome Brain; Lessons from Alzheimer’s Disease. Curr. Gerontol. Geriatr. Res..

[52] Luque-Martin R., Angell D.C., Kalxdorf M., Bernard S., Thompson W., Eberl H.C., Ashby C., Freudenberg J., Sharp C., Van den Bossche J. (2021). IFN-γ Drives Human Monocyte Differentiation into Highly Proinflammatory Macrophages That Resemble a Phenotype Relevant to Psoriasis. J. Immunol..

[53] Zhou J., Zhang J., Tao L., Peng K., Zhang Q., Yan K., Luan J., Pan J., Su X., Sun J. (2022). Up-Regulation of BTN3A1 on CD14+ Cells Promotes Vγ9Vδ2 T Cell Activation in Psoriasis. Proc. Natl. Acad. Sci. USA.

[54] He J., Zhao M., Ma X., Li D., Kong J., Yang F. (2023). The Role and Application of Three IFN-Related Reactions in Psoriasis. Biomed. Pharmacother..

[55] Schnell A., Huang L., Singer M., Singaraju A., Barilla R.M., Regan B.M.L., Bollhagen A., Thakore P.I., Dionne D., Delorey T.M. (2021). Stem-like Intestinal Th17 Cells Give Rise to Pathogenic Effector T Cells during Autoimmunity. Cell.

[56] Manivasagam S., Klein R.S. (2021). Type III Interferons: Emerging Roles in Autoimmunity. Front. Immunol..

[57] Cai Y.-X., Zheng D.-S., Chen X.-L., Bai Z.-P., Zhang J., Deng W., Huang X.-F. (2025). An Integrated Multi-Omics Analysis Identifies Protein Biomarkers and Potential Drug Targets for Psoriatic Arthritis. Commun. Biol..

[58] Deprince A., Hennuyer N., Kooijman S., Pronk A.C.M., Baugé E., Lienard V., Verrijken A., Dirinck E., Vonghia L., Woitrain E. (2023). Apolipoprotein F Is Reduced in Humans with Steatosis and Controls Plasma Triglyceride-rich Lipoprotein Metabolism. Hepatology.

[59] Xie W., Li J., Du H., Xia J. (2023). Causal Relationship between PCSK9 Inhibitor and Autoimmune Diseases: A Drug Target Mendelian Randomization Study. Arthritis Res. Ther..

[60] Liu M., Chen M., Tan J., Chen A., Guo J. (2024). Plasma Proteins and Inflammatory Dermatoses: Proteome-Wide Mendelian Randomization and Colocalization Analyses. Arch. Dermatol. Res..

[61] Saxe J.P., Chen M., Zhao H., Lin H. (2013). Tdrkh Is Essential for Spermatogenesis and Participates in Primary piRNA Biogenesis in the Germline. EMBO J..

[62] Sun X., Wu B., Chiang H.-C., Deng H., Zhang X., Xiong W., Liu J., Rozeboom A.M., Harris B.T., Blommaert E. (2021). Tumour DDR1 Promotes Collagen Fibre Alignment to Instigate Immune Exclusion. Nature.

[63] Wynford-Thomas R., Jacob A., Tomassini V. (2019). Neurological Update: MOG Antibody Disease. J. Neurol..

[64] Thakker P., Leach M.W., Kuang W., Benoit S.E., Leonard J.P., Marusic S. (2007). IL-23 Is Critical in the Induction but Not in the Effector Phase of Experimental Autoimmune Encephalomyelitis. J. Immunol..

[65] Shen W., Xie J., Zhao S., Du R., Luo X., He H., Jiang S., Hao N., Chen C., Guo C. (2018). ICAM3 Mediates Inflammatory Signaling to Promote Cancer Cell Stemness. Cancer Lett..

[66] Salminen A.T., Tithof J., Izhiman Y., Masters E.A., McCloskey M.C., Gaborski T.R., Kelley D.H., Pietropaoli A.P., Waugh R.E., McGrath J.L. (2020). Endothelial Cell Apicobasal Polarity Coordinates Distinct Responses to Luminally versus Abluminally Delivered TNF-α in a Microvascular Mimetic. Integr. Biol..

[67] Kang S., Kim J., Park A., Koh M., Shin W., Park G., Lee T.A., Kim H.J., Han H., Kim Y. (2023). TRIM40 Is a Pathogenic Driver of Inflammatory Bowel Disease Subverting Intestinal Barrier Integrity. Nat. Commun..

