# Supplementary material for: Integrative Analysis of Plasma Proteomics and Transcriptomics Reveals Potential Therapeutic Targets for Psoriasis
Source: Biomedicines. 2025 Jun 4;13(6):1380. doi: 10.3390/biomedicines13061380 (PMC12190617; doi:10.3390/biomedicines13061380)
Supplement: Supplementary file 1 [file biomedicines-13-01380-s001.zip › Supplementary Figure.pdf]

Supplementary Figures

Supplementary Figure S1. Horizontal pleiotropy may result in false causality, leading to ineffective drug targets.

Supplementary Figure S2. The association plot of the colocalization region between APOF and psoriasis.

Supplementary Figure S3. The association plot of the colocalization region between DDR1 and psoriasis.

Supplementary Figure S4. The association plot of the colocalization region between HCG22 and psoriasis.

Supplementary Figure S5. The association plot of the colocalization region between ICAM3 and psoriasis.

Supplementary Figure S6. The association plot of the colocalization region between IFNGR2 and psoriasis.

Supplementary Figure S7. The association plot of the colocalization region between IFNLR1 and psoriasis.

Supplementary Figure S8. The association plot of the colocalization region between IL12B and psoriasis.

Supplementary Figure S9. The association plot of the colocalization region between MOG and psoriasis.

Supplementary Figure S10. The association plot of the colocalization region between TDRKH and psoriasis.

Supplementary Figure S11. The association plot of the colocalization region between BTN3A2 and psoriasis.

Supplementary Figure S12. The association plot of the colocalization region between LTA and psoriasis.

Supplementary Figure S13. Single-cell expression of protein-coding genes in normal skin samples.

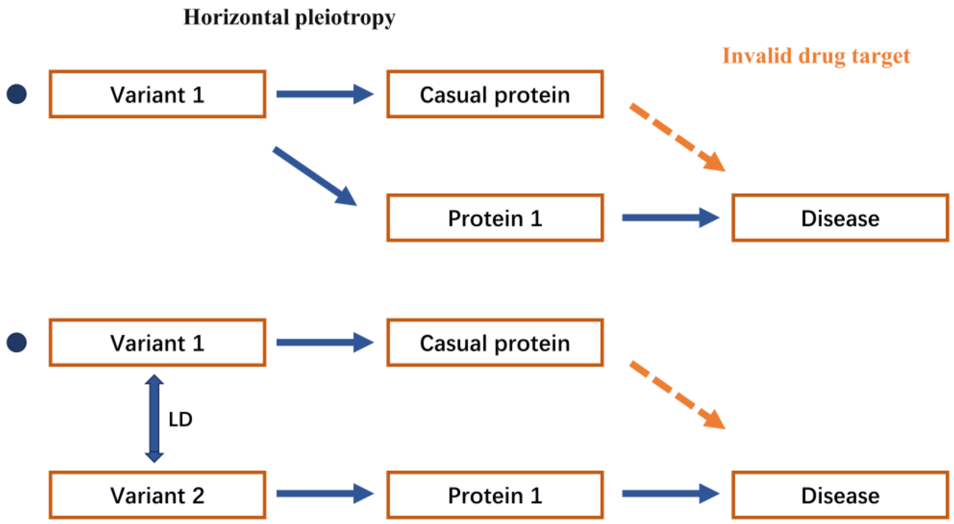

Supplementary Figure S1. Horizontal pleiotropy may result in false causality, leading to ineffective drug targets. False causality between the identified causal protein and the disease may arise from the genetic variant itself or from linkage disequilibrium (LD) of the variant with other proteins (Protein 1)

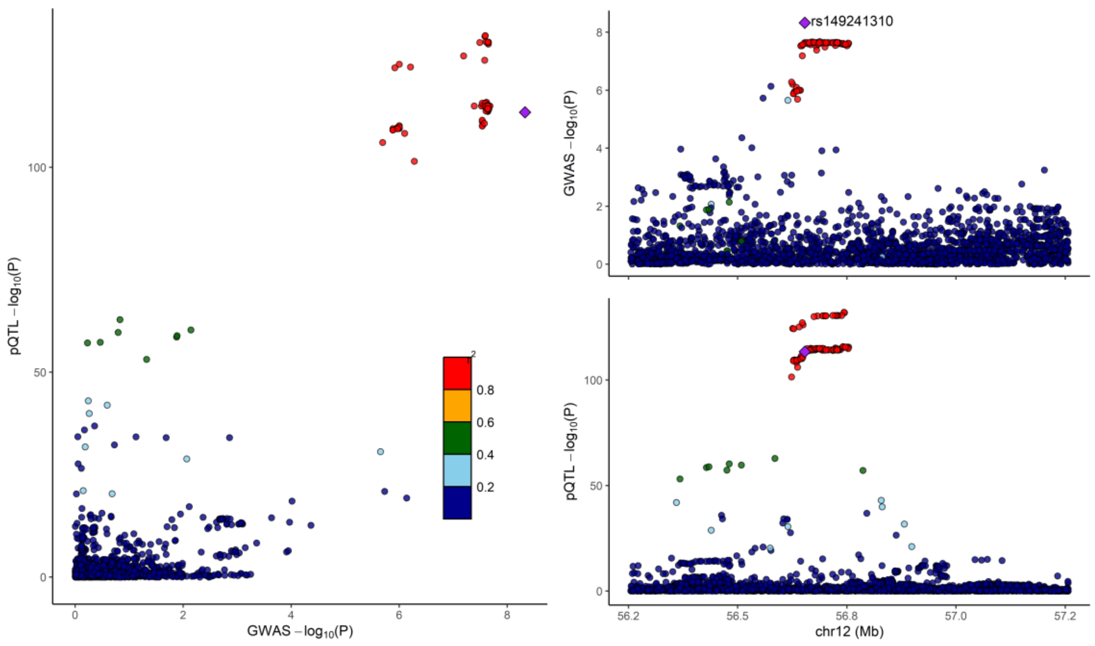

Supplementary Figure S2. The association plot of the colocalization region between APOF and psoriasis.

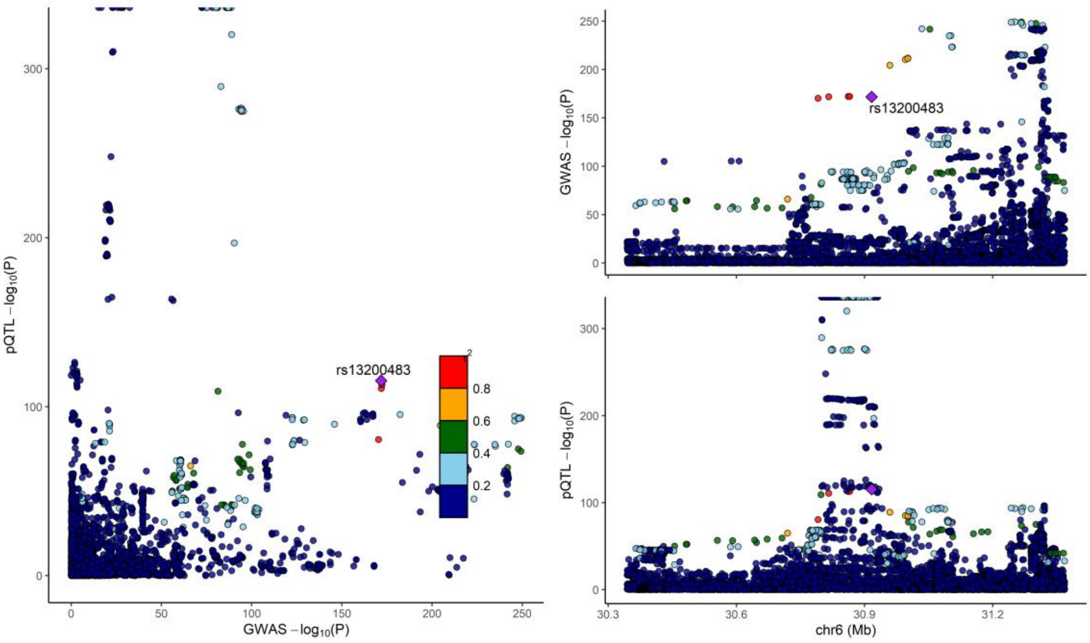

Supplementary Figure S3. The association plot of the colocalization region between DDR1 and psoriasis.

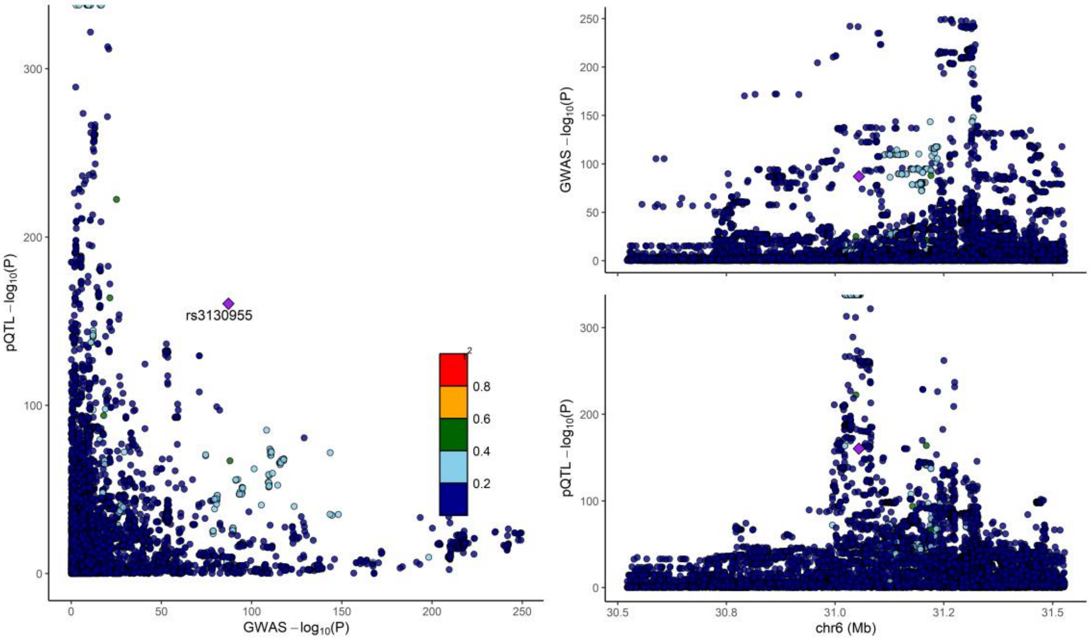

Supplementary Figure S4. The association plot of the colocalization region between HCG22 and psoriasis.

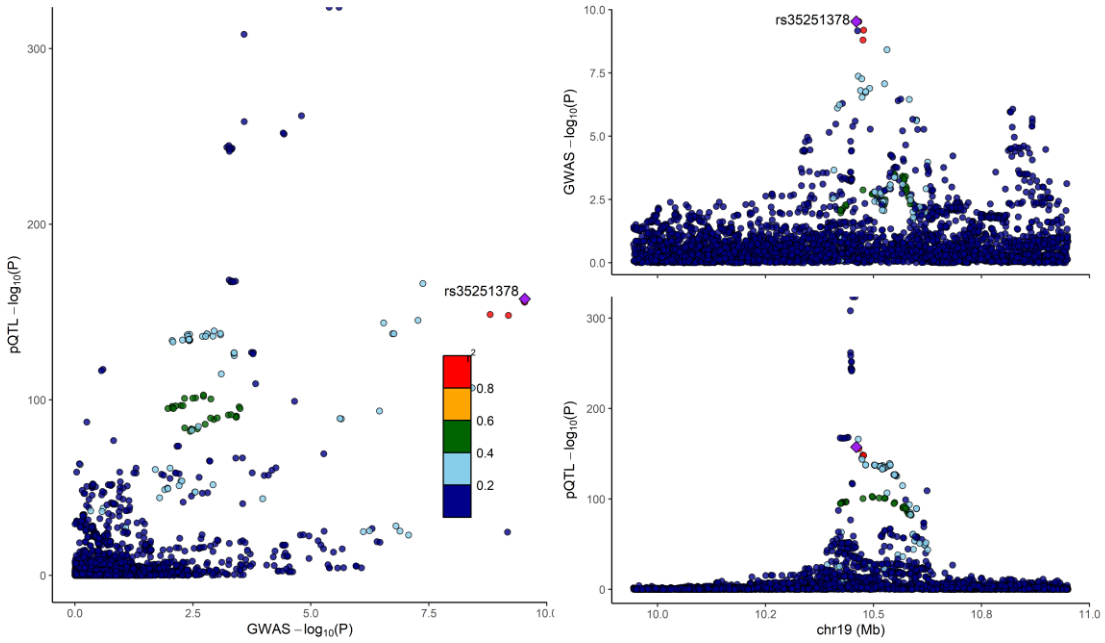

Supplementary Figure S5. The association plot of the colocalization region between ICAM3 and psoriasis.

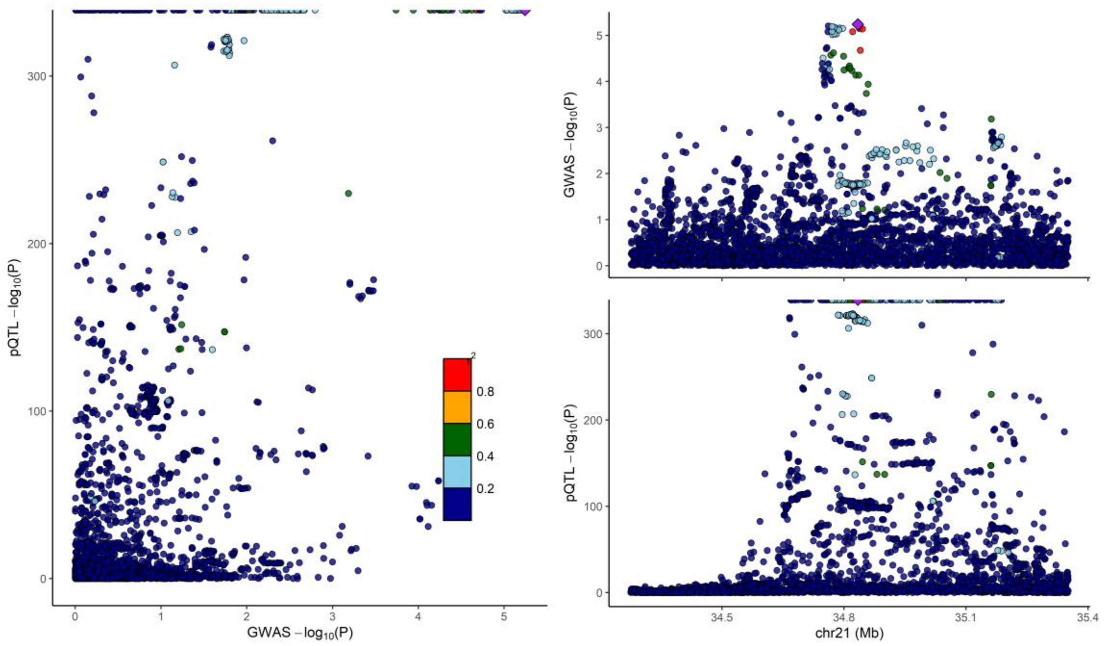

Supplementary Figure S6. The association plot of the colocalization region between IFNGR2 and psoriasis.

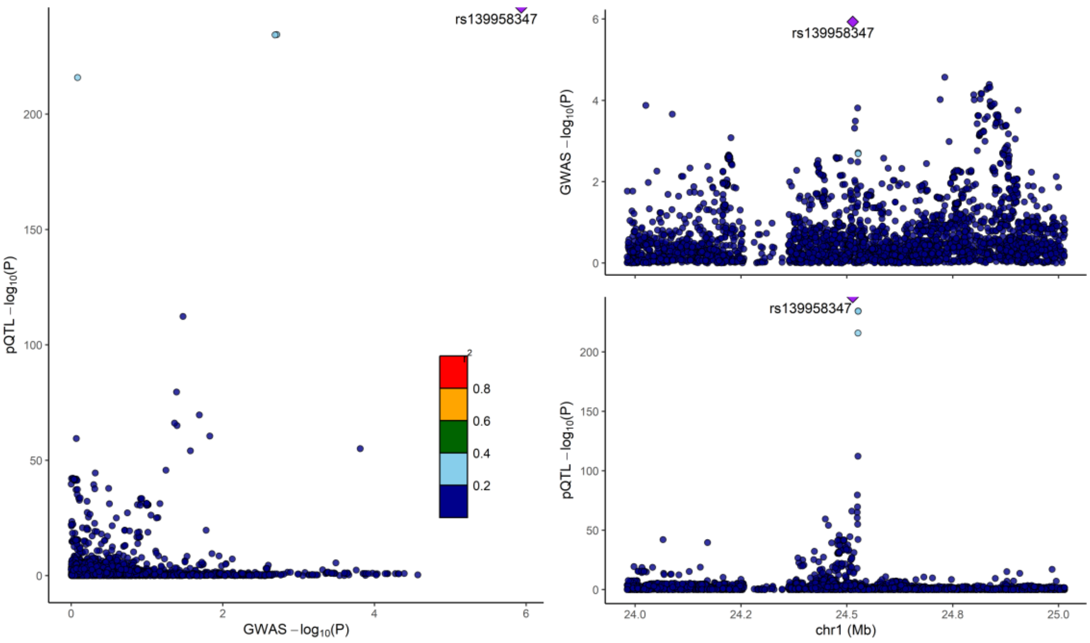

Supplementary Figure S7. The association plot of the colocalization region between IFNLR1 and psoriasis.

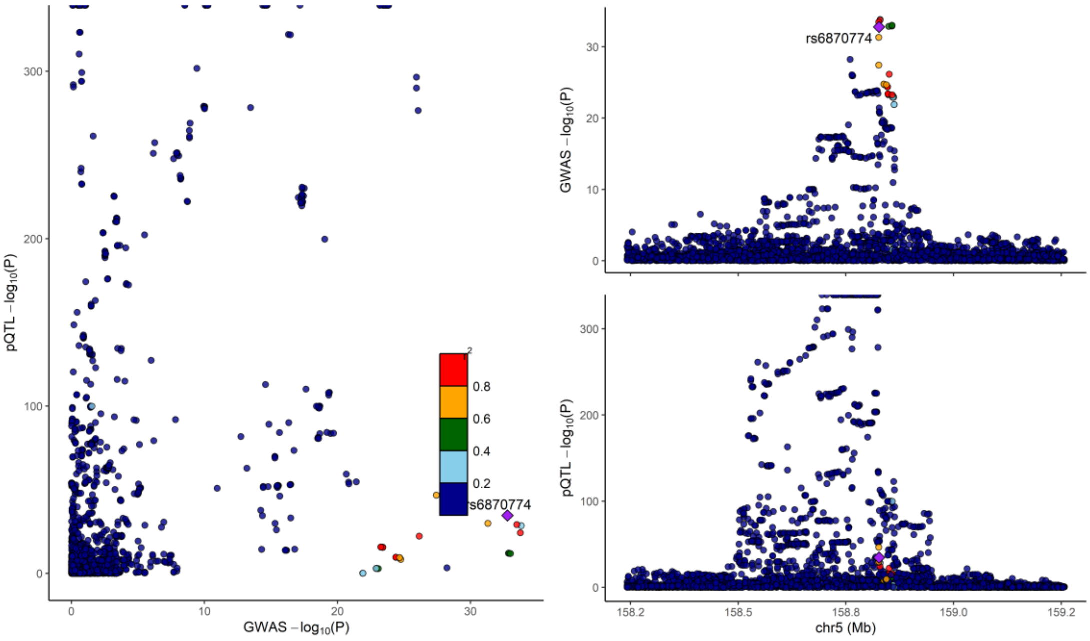

Supplementary Figure S8. The association plot of the colocalization region between IL12B and psoriasis.

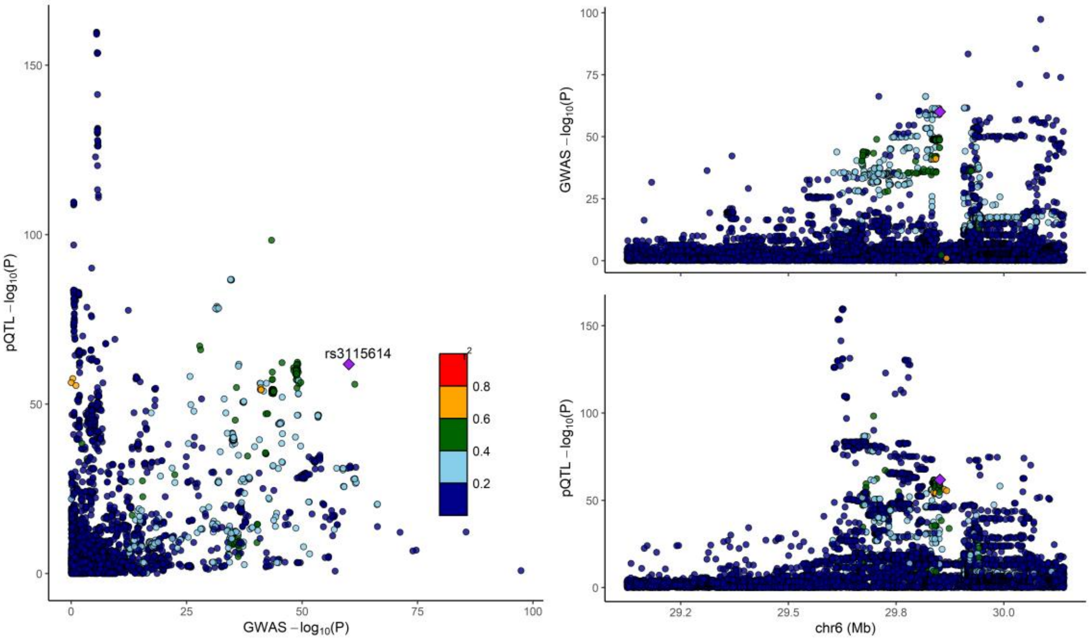

Supplementary Figure S9. The association plot of the colocalization region between MOG and psoriasis.

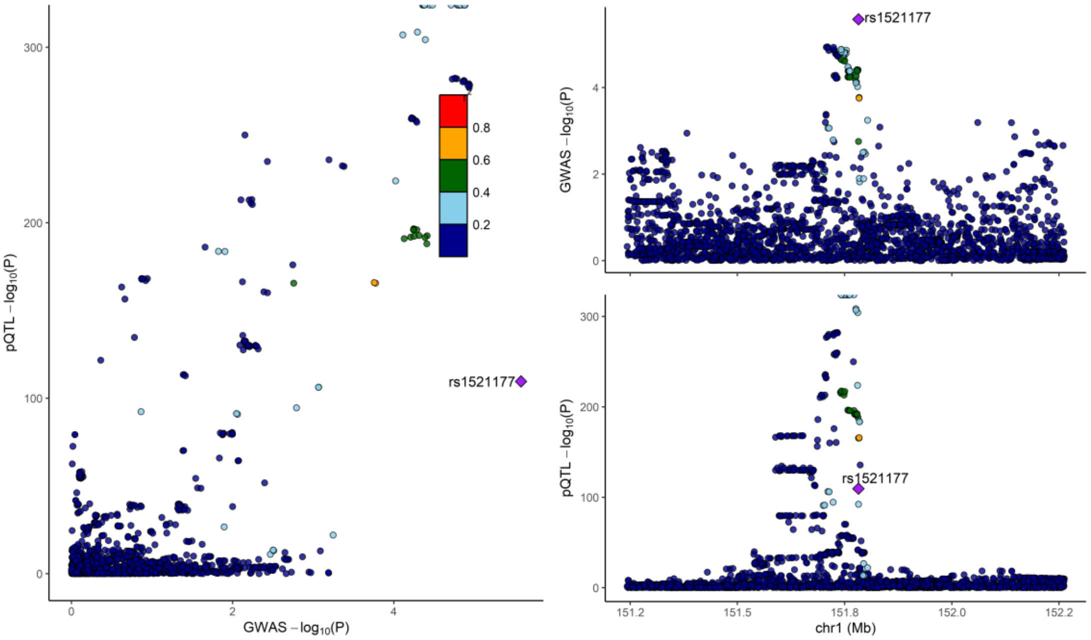

Supplementary Figure S10. The association plot of the colocalization region between TDRKH and psoriasis.

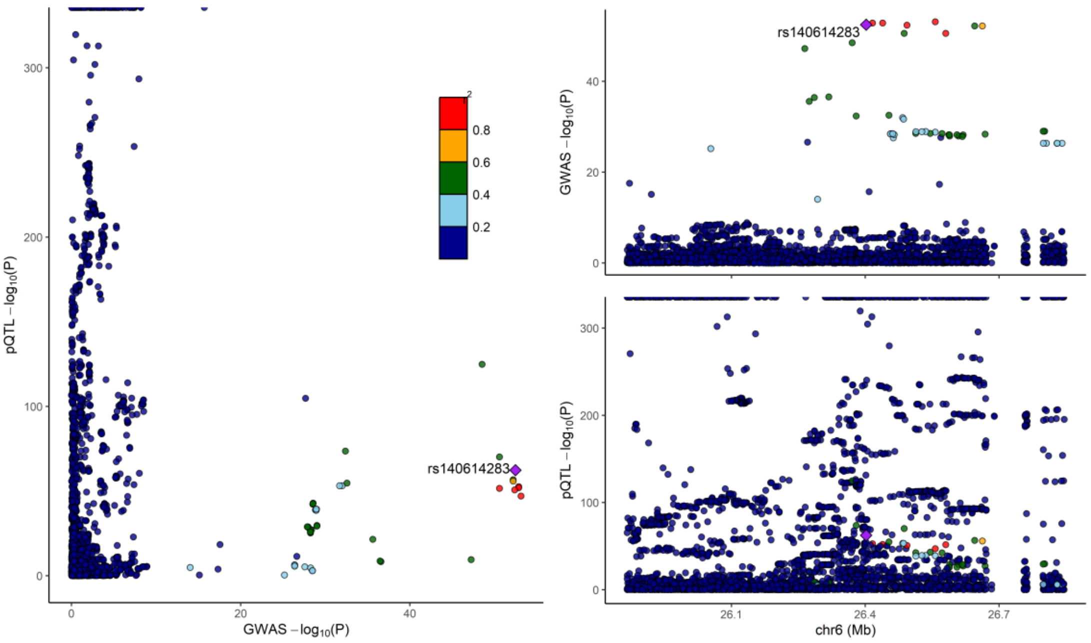

Supplementary Figure S11. The association plot of the colocalization region between BTN3A2 and psoriasis.

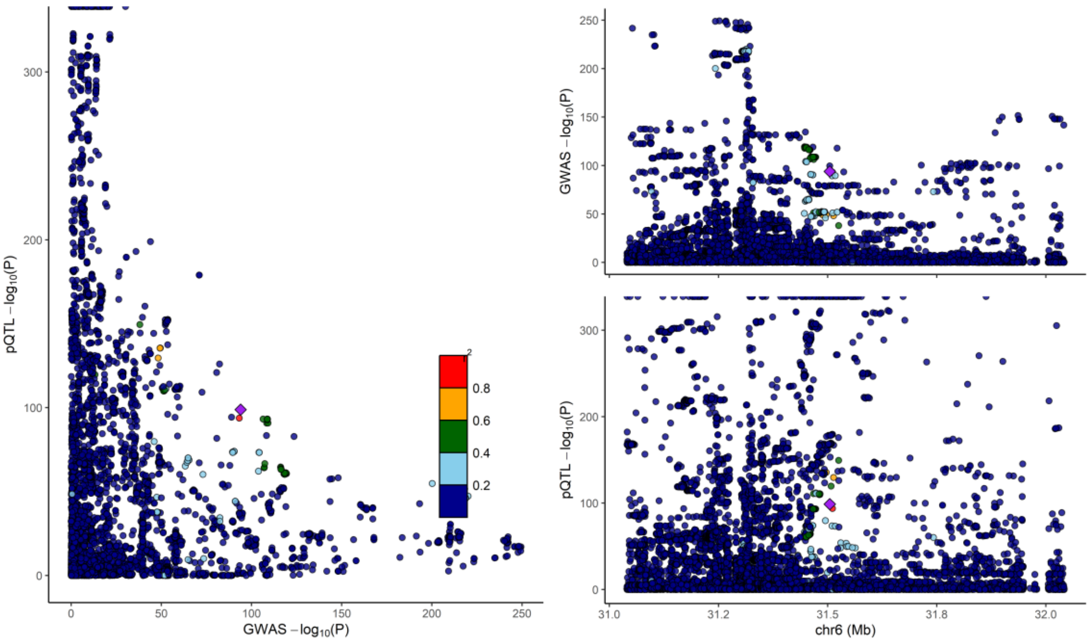

Supplementary Figure S12. The association plot of the colocalization region between LTA and psoriasis.

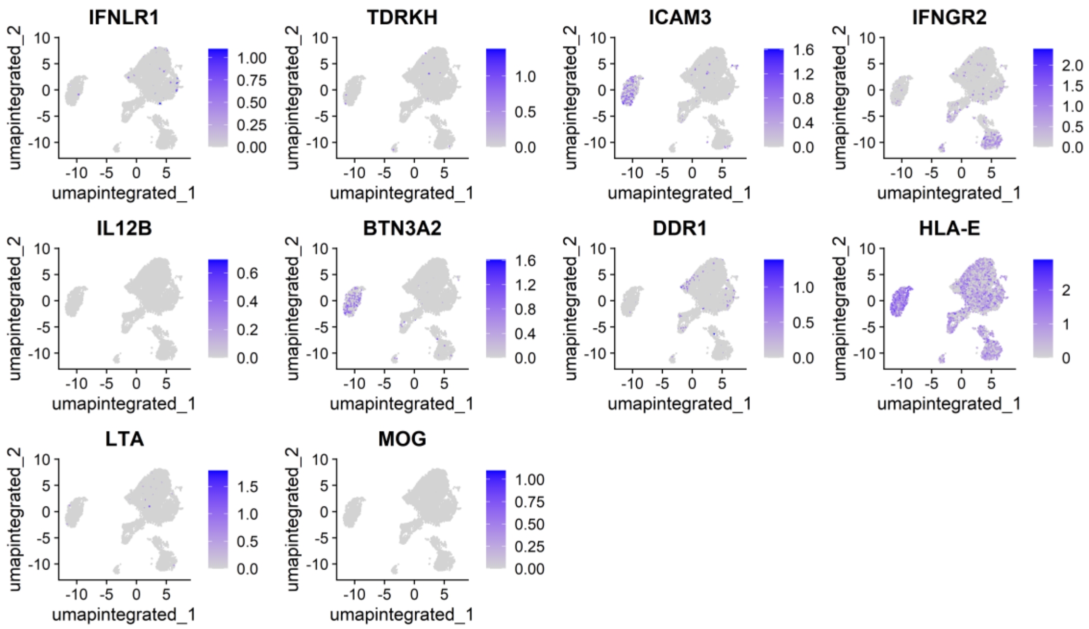

Supplementary Figure S13. Single-cell expression of protein-coding genes in normal skin samples.
